# Effect of 4-Week Consumption of “Navelina” Oranges on Serum Lipid Profile in Patients with MASLD: Evidence from a Randomized Clinical Trial

**DOI:** 10.3390/nu18081254

**Published:** 2026-04-16

**Authors:** Valentina De Nunzio, Giuliano Pinto, Davide Guido, Emanuela Aloisio Caruso, Miriam Cofano, Ilenia Saponara, Matteo Centonze, Maria Grazia Refolo, Maria Notarnicola

**Affiliations:** 1 Laboratory of Nutritional Biochemistry, National Institute of Gastroenterology IRCCS “Saverio de Bellis”, 70013 Castellana Grotte, Italy; valentina.denunzio@irccsdebellis.it (V.D.N.); giuliano.pinto@irccsdebellis.it (G.P.); emanuela.caruso@irccsdebellis.it (E.A.C.); miriam.cofano@irccsdebellis.it (M.C.); ilenia.saponara@irccsdebellis.it (I.S.); matteo.centonze@irccsdebellis.it (M.C.); 2 Data Science Unit, National Institute of Gastroenterology IRCCS “Saverio de Bellis”, 70013 Castellana Grotte, Italy; davide.guido@irccsdebellis.it; 3 Laboratory of Clinical Pathology, National Institute of Gastroenterology IRCCS “Saverio de Bellis”, 70013 Castellana Grotte, Italy; maria.refolo@irccsdebellis.it

**Keywords:** MASLD, serum lipid profile, lipidomic analysis, Oleic acid, AA/EPA ratio, *n*-3 PUFAs, HDL

## Abstract

**Background:** Metabolic Dysfunction-Associated Steatotic Liver Disease (MASLD) refers to fatty liver disease associated with metabolic syndrome. MASLD causes alterations in lipid metabolism, which can be regulated with a diet rich in polyphenols. The present study aims to evaluate the effects of daily consumption of 400 g of “Navelina” oranges for 4 weeks on serum lipid profiles in a group of 60 patients with MASLD, to identify specific lipid species associated with improvements in hepatic steatosis. **Methods:** Blood samples were collected from all participants, and biochemical measurements and a serum lipidomic profile were performed. Finally, a Spearman correlation analysis was used to assess the relationships between serum lipidomic fatty acids and biochemical lipid markers. **Results:** In the experimental treatment arm, serum lipidomic analysis showed a slight decrease in Arachidonic acid (AA) and the Arachidonic acid/Eicosapentaenoic acid ratio (AA/EPA ratio) but no significant interaction between time and treatment was detected. In the same group, Oleic acid, MUFAs and the AA/EPA ratio were significantly and negatively correlated with HDL (r = −0.368, *p* = 0.046), (r = −0.384, *p* = 0.036), and (r = −0.522, *p* = 0.003), respectively. Conversely, EPA and *n*-3 PUFAs were positively and significantly correlated with HDL (r = 0.447, *p* = 0.013) and (r = 0.403, *p* = 0.027) respectively. **Conclusions:** Furthermore, this study represents one of the first clinical trials to shed a light on the potential association of “Navelina” orange polyphenols on serum fatty acid profiles in patients with MASLD, supporting studies on the nutraceutical effect of oranges on lipid metabolism.

## 1. Introduction

Metabolic Dysfunction-Associated Steatotic Liver Disease (MASLD), previously referred to as non-alcoholic fatty liver disease (NAFLD), denotes fatty liver disease associated with metabolic syndrome and is strongly linked to visceral obesity, type 2 diabetes mellitus, and other cardiometabolic risk factors [1,2,3]. MASLD currently represents one of the leading causes of liver-related morbidity, mortality, and liver transplantation worldwide [4]. To date, lifestyle modification remains the most effective therapeutic strategy for MASLD, with dietary interventions and increased physical activity as central components [5,6].

In MASLD, profound alterations in lipid metabolism are frequently observed [7]. Plasma and tissue fatty acids are predominantly present in esterified forms within triglycerides, phospholipids, and cholesterol esters, constituting a dynamic system involved in energy transport, cellular structure, and metabolic regulation. Cholesterol esters represent a major storage and transport form of cholesterol within circulating lipoproteins, particularly low-density lipoproteins (LDL) and high-density lipoproteins (HDL). LDL-associated cholesterol contributes to vascular accumulation, whereas HDL mediates reverse cholesterol transport, exerting a protective role against atherosclerosis [7,8,9]. HDL removes excess cholesterol from peripheral tissues, which is why it is called “good cholesterol.” A healthy lifestyle and a balanced diet increase serum HDL level, improving an individual’s lipid profile [7].

Lipidomics is an omics-based approach dedicated to the comprehensive characterization of lipid profiles in biological samples, including plasma, serum, urine, and tissue specimens [10]. From a methodological standpoint, lipidomics relies on highly sensitive analytical techniques such as gas chromatography (GC) coupled with flame ionization detection (FID) or mass spectrometry (MS), as well as liquid chromatography (LC) coupled with MS. These technologies enable the separation, identification, and quantification of hundreds of lipid species within a single biological sample [11].

Lipids are extracted from blood or other biological fluids and subjected to specific derivatization procedures, allowing not only the measurement of total fatty acid content but also the determination of their distribution among major lipid classes, including saturated fatty acids (SFAs), monounsaturated fatty acids (MUFAs), and polyunsaturated fatty acids (PUFAs) [11]. This approach ultimately provides a comprehensive lipidomic profile that reflects the metabolic status of the organism.

Each lipid class has a distinct biological role; some function as energy reserves, others serve as essential structural components of cellular membranes, while others act as signaling molecules regulating key processes such as inflammation, programmed cell death, and immune responses [12,13]. SFAs, including Palmitic and Stearic acids, are frequently associated with hepatic lipotoxicity, increased inflammation, and insulin resistance [14,15]. Conversely, MUFAs, particularly Oleic acid, exert protective effects on lipid and glucose metabolism by promoting improved insulin sensitivity [16]. PUFAs are classified into two main families: omega-3 (*n*-3) and omega-6 (*n*-6). The *n*-3 family includes alpha-Linolenic acid (ALA), Eicosapentaenoic acid (EPA), and Docosahexaenoic acid (DHA), whereas the *n*-6 family includes Linoleic acid (LA) and Arachidonic acid (AA). PUFAs, especially those belonging to the *n*-3 family, display potent anti-inflammatory properties and modulate gene expression involved in lipid oxidation and triglyceride synthesis. ALA and LA are considered essential fatty acids because they cannot be synthesized by humans and must therefore be obtained through dietary sources. Through the coordinated action of elongases and desaturases, these fatty acids give rise to the respective *n*-3 and *n*-6 metabolic cascades [17].

Consequently, the lipidomic profile represents a true “metabolic snapshot,” capable of revealing biochemical imbalances even before the onset of clinical symptoms. Accordingly, lipidomics is gaining increasing importance in the identification of novel lipid biomarkers in metabolic, cardiovascular, neurodegenerative, and oncological diseases [18].

Serum fatty acids also serve as biological biomarkers of dietary intake, highlighting the close interconnection between lipidomics and nutrition. Dietary lipids directly enter metabolic pathways and are incorporated into tissues. Diets rich in saturated and trans fatty acids are associated with increased membrane rigidity and chronic inflammation, thereby enhancing the risk of metabolic syndrome and cardiovascular disease [19]. Conversely, adequate PUFAs intake contributes to the modulation of inflammatory processes. Balanced diets rich in PUFAs, particularly *n*-3 fatty acids derived from fish, nuts, and vegetable oils, promote a favorable lipidomic profile, exerting anti-inflammatory and cardioprotective effects. Recent lipidomic studies have also identified specific phosphatidylcholine species as surrogate biomarkers of the *n*-3 index, further highlighting the potential of lipidomics for personalized nutritional assessment [20].

The lipid profile is also modulated by other dietary bioactive compounds, such as polyphenols. Polyphenols are phenolic compounds widely present in fruits, vegetables, cereals, legumes, olives, chocolate, tea, coffee, wine, and grape pomace [21]. Diets rich in polyphenols have been associated with improvements in lipid metabolism, enhanced insulin sensitivity, and reduced metabolic risk. Their protective role in human health has been extensively documented, including their capacity to modulate gastrointestinal function and inflammatory responses [21,22].

Notably, grape polyphenols have been shown to influence cellular membrane fluidity and cell motility through modulation of stearoyl-CoA desaturase-1 (SCD1) activity, a key enzyme involved in the conversion of Stearic acid to Oleic acid, as reflected by the Oleic/Stearic acid ratio [23]. Similarly, hesperidin, the major polyphenol found in “Navelina” oranges, has been reported to modulate SCD1 expression. An in vitro study demonstrated that hesperidin reduced SCD1 expression in steatotic HEPA-RG cells, contributing to membrane stabilization and attenuation of oxidative stress [24].

In our previous study involving a cohort of patients with MASLD, we found that supplementing the habitual diet with 400 g/day of “Navelina” oranges for 4 weeks resulted in a variation in hepatic steatosis. This occurred despite the absence of changes in body weight, waist circumference, body composition, and conventional lipid profile parameters [5].

Based on these previously published findings, this study aims to evaluate the effects of daily consumption of “Navelina” oranges on the serum lipid profile in patients with MASLD by identifying specific lipid species.

## 2. Materials and Methods

### 2.1. Participants

Sixty subjects (43 men, 71.67%) aged 30–65 years diagnosed with MASLD were recruited from the nutrition clinic of the National Institute of Gastroenterology IRCCS “S. de Bellis” between February 2023 and November 2023. The inclusion criteria required participants to have fatty liver disease (Controlled Attenuation Parameter, CAP score > 275 dB/m) together with overweight (Body Mass Index, BMI > 25), type 2 diabetes and/or metabolic syndrome as previously described [1]. Approval for this study was obtained from the Human Studies Committee of the IRCCS Oncological Hospital—Giovanni Paolo II, Bari, Italy (Approval Number #184 del 13 May 2022).

All participants provided informed consent following the principles outlined in the Declaration of Helsinki. This clinical trial was registered at ClinicalTrials.gov (NCT05558592).

Participants were randomly assigned to two study arms (control arm and experimental treatment arm) using a computer-generated sequence of non-unique, unordered numbers between 1 and 2. Briefly, the experimental treatment arm subjects were invited to consume 400 g of “Navelina” orange daily for 4 weeks. All enrolled subjects received dietary recommendations, such as limitation of alcohol, caffeine, and polyphenol-rich foods. The participants assigned to the control arm were asked to abstain from eating oranges. At baseline and after 4 weeks of orange treatment, all subjects, including the controls, were asked to undergo a blood draw for fatty acids evaluation and biochemical analyses. All participants completed a self-report adherence questionnaire and food diary one week before and throughout the trial.

### 2.2. Blood Samples

Blood samples were collected from all participants after a 12 h fast in tubes containing silica gel as a clotting activator at baseline and after 4 weeks (follow-up). Serum samples were separated by centrifugation at 3200 rpm for 10 min and used appropriately.

### 2.3. Biochemical Measurements

Biochemical measurements were performed at the Laboratory of Clinical Pathology of our Institute. Total cholesterol, HDL, and LDL were assayed by sets of Cobas 8000 (Roche Diagnostics S.p.A., Monza, Italy) auto-analyzers.

### 2.4. sdLDL Analysis

sdLDL were measured using the Lipoprint LDL system (Quantimetrix, Redondo Beach, CA, USA), an electrophoresis system capable of separating lipoprotein fractions and subfractions.

Briefly, 25 µL of serum with 200 µL of Lipoprint Loading Gel (containing Sudan Black B dye to stain lipoproteins) were loaded into 3% polyacrylamide gel tubes. After 30 min at room temperature, the electrophoresis run started for 1 h. At the end of the run, to increase band uniformity, the samples were left at room temperature for 30 min. The lipoprotein bands obtained were scanned, and the generated images were analyzed using Lipoware software(V1.95 year 2003).

### 2.5. Serum Fatty Acid Extraction and Analysis

Fatty acid extraction from serum was performed using the Fatty Acid Extraction Kit, Low Standard (Sigma-Aldrich, St. Louis, MO, USA) according to the manufacturer’s instructions.

Briefly, 200 μL of serum per sample was treated with 3 mL of extraction solvent and 450 μL of aqueous buffer, vortexed, and placed in a syringe to elute the lipids. The eluted lipids were dried and esterified with a methanolic potassium hydroxide solution and sonicated for 30 min. 1 mL of hexane and 1 mL of distilled water were added to the extracted mixture. The top of the hexane layer was transferred to a new tube and dried. Esterified lipids were reconstituted with 100 μL hexane and analyzed using a gas chromatography Agilent 8890 GC System (AGILENT, Milan, Italy) apparatus with an autosampler, split/splitless injector, FID detector, and hydrogen generator (Cinel gas generation AD-180, Padua, Italy). The analysis was performed on a BPX 70 capillary column (SGE Analytical Science, P/N SGE054623, 60 m × 0.25 mm ID—BPX70 0.25 μM, SGE Europe Ltd., Milton Keynes, UK). Hydrogen was used as the carrier gas, 3.0 mL min^−1^, in constant flow mode; the injected amount was 1 μL in splitless mode (split flow rate 50 mL min^−1^, splitless time 1 min). The injector and FID detector temperatures were 250 °C and 270 °C, respectively. The initial oven temperature was 40 °C, then increased to 170 °C at 10 °C min^−1^ for 5 min, then to 200 °C at 4 °C min^−1^ for another 5 min, and finally the temperature was increased to 255 °C at 50 °C min^−1^ and held for 4.5 min. Each peak was identified by comparing retention times with those of a standard mixture (Supelco 37-Component FAME Mix, Sigma-Aldrich, Milan, Italy).

Data were expressed as the percentage of each fatty acid calculated from the total amount of fatty acids.

### 2.6. Statistical Analysis

Data are expressed as median and interquartile range (IQR) or mean ± standard deviations for continuous variables, and frequencies and percentages for the categorical ones. Firstly, associations between treatments and variables at baseline were tested by the non-parametric Mann–Whitney test or Chi-squared test (or Fisher’s exact test), as appropriate.

#### 2.6.1. GEE Modeling

Subsequently, generalized estimating equation (GEE) models [25,26] for repeated measures were applied to assess the differences in lipid, biological and clinical markers among individuals across treatment and time. For each outcome, we fitted a GEE model where time—considered as a categorical two-level factor (i.e., baseline as reference, and at follow-up, after 4 weeks)—treatment (i.e., experimental treatment vs. control one, by using the latter as reference), and their interaction terms were ‘‘focus’’ predictors. Gamma GEE models were fitted to manage the skewed continuous nature by using the “identity’’ link, an exchangeable correlation structure, and a sandwich robust estimation, to consider model misspecifications. In addition, Gaussian GEE models were fitted on outcomes assuming null values. Briefly, the random variable Gamma has probabilistic support considering positive values (i.e., y > 0), whereas the random variable Gaussian has a probabilistic support on real numbers (i.e., −Inf, +Inf).

GEE models were chosen for their robustness and because they allowed the management of the intra-subject variability produced by two separate measurements carried out on the same patients (n = 30 × 2 = 60 observations per arm, but only 30 independent) [27]. The models’ time parameters (β_T_) were interpreted as average time changes of the outcomes between the two separate measurements (follow-up—baseline measurements); the parameters associated with the treatment (β_TR_) were interpreted as average baseline differences between the two treatments (the control one represented the reference category). Finally, the interaction parameters (β_TxTR_) were interpreted as average time differences of the outcomes (from baseline) between treatments. Estimates were adjusted for baseline values of total and HDL cholesterol, dietary lipids, and intakes of proteins and carbohydrates to manage imbalances across treatments by avoiding the risk of confounding.

#### 2.6.2. Correlation Analysis

Finally, a Spearman correlation analysis was used to assess the relationships among serum lipidomic fatty acids, such as Oleic acid, EPA, MUFAs, *n*-3 PUFAs and AA/EPA ratio, as well as biochemical markers (total cholesterol, LDL and HDL) after 4 weeks (follow-up–baseline) in the arm-stratified samples.

The Spearman correlation coefficient (r) is typically used to evaluate the raw association between two variables in a non-parametric framework (i.e., lack of normality). The value of r covers the whole range of relationship strengths, from no relationship whatsoever (r = 0) to a perfect relationship (r = 1 or r = −1) [28]. Cohen provided guidelines for interpreting these effect sizes, suggesting that an r of |0.1| represents a “small” effect size, |0.3| represents a “medium” effect size, while |0.5| represents a “large” effect size [29,30].

Wald tests and 95% confidence intervals were carried out to evaluate statistical significance on model parameters. Two-tailed *p*-values < 0.05 were considered significant. Calculations were carried out using the statistical software R (version 4.3.3) (R Core Team, 2024) and its package geepack [25,31,32], Hmisc [33], pwr [34] and ggplot2 [35].

## 3. Results

Table 1 shows demographic data, anthropometric measurements, weekly dietary intake and biochemical parameters at baseline.

Adherence in both arms was high, with overall compliance rates of 96.8% and 93.1% in the experimental treatment arm and control arm, respectively. Although the bioavailability of polyphenols was not measured, we can hypothesize that the data can be attributed to “Navelina” orange since all participants avoided consumption of other citrus fruits or similar foods.

Regarding the GEE modeling, Table 2 outlines the results in terms of coefficients, *p*-values, and 95%CIs. No significant time-treatment interaction was observed across the modeling; in other words, no experimental treatment effect (in relation to the control arm) was significant across time. However, the GEE analysis revealed a downward trend in the interaction term (β_TxTR_) for total cholesterol (*p*-value = 0.060).

However, there was a slight reduction in AA and the AA/EPA ratio in the experimental treatment arm. It is also interesting to note that cholesterol, LDL, and HDL values underwent changes. In particular, there was a decrease in total cholesterol and LDL levels, alongside an increase in HDL levels following the consumption of oranges; however, these changes were not significant.

Table 3 reports the results of the Spearman correlation (r) analysis of the Δ-changes (follow-up—baseline) of serum fatty acids and biochemical lipid markers by arm. In the experimental treatment arm, Oleic acid, MUFAs, and the AA/EPA ratio were significantly negatively correlated with HDL (r = −0.368, *p* = 0.046), (r = −0.384, *p* = 0.036), and (r = −0.522, *p* = 0.003), respectively. A positive and significant correlation was also found between EPA vs. total cholesterol (r = 0.386, *p* = 0.035) and HDL (r = 0.447, *p* = 0.013) and between *n*-3 PUFA vs. HDL (r = 0.403, *p* = 0.027). In the control group, negative and statistically significant correlations were found between Oleic acid (r = −0.463, *p* = 0.010) and MUFAs (r = −0.402, *p* = 0.028) vs. total cholesterol.

**Figure 1:**
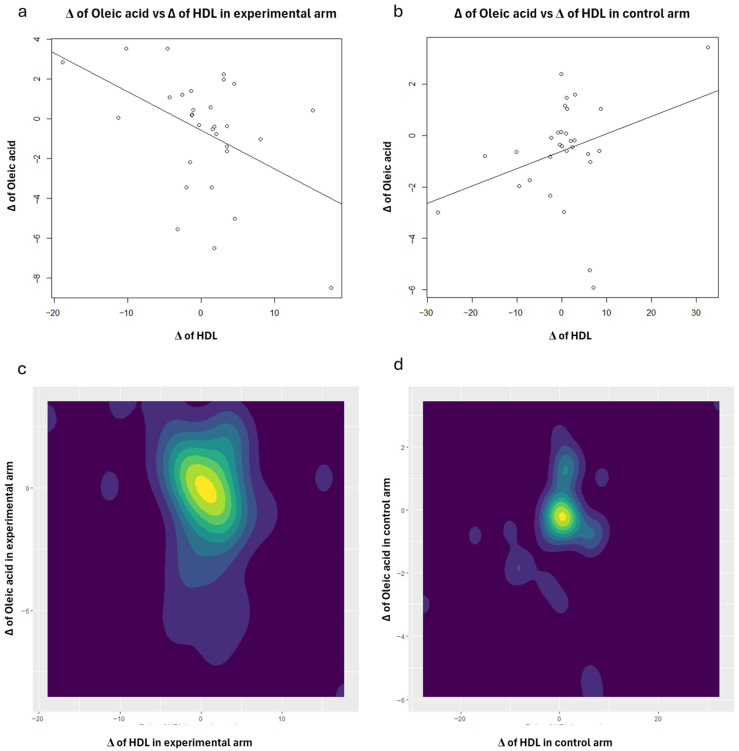
Scatter plots: joint distributions and regression lines of Δ-changes of HDL (*x*-axis) and Oleic acid, within experimental treatment arm (**a**) and control arm (**b**). Contour plots: joint distributions of Δ-changes of HDL (*x*-axis) and Oleic acid, within experimental treatment arm (**c**) and control arm (**d**).

**Figure 2:**
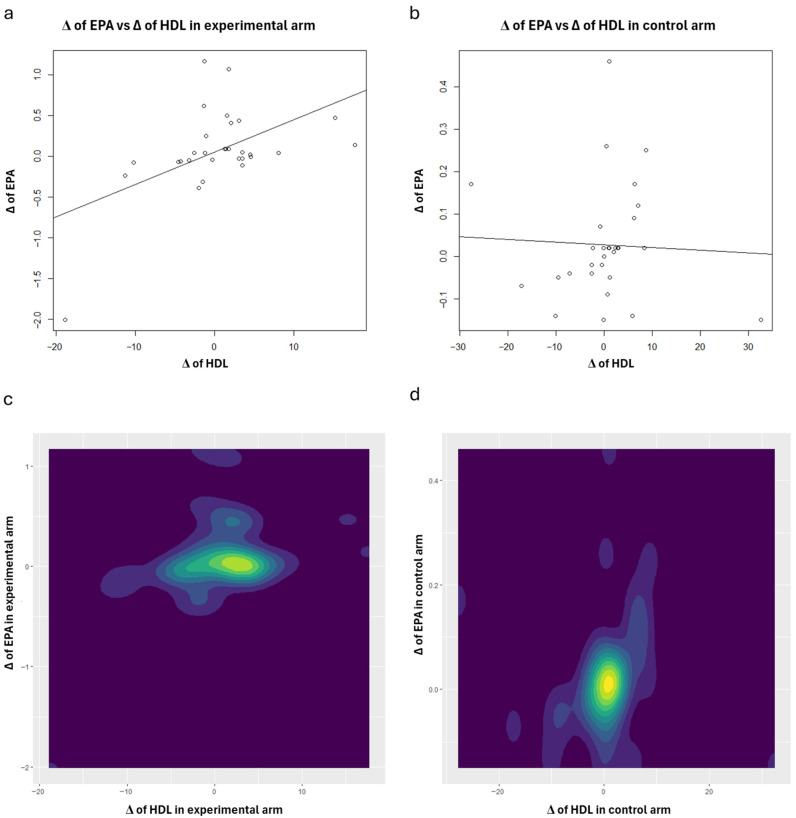
Scatter plots: joint distributions and regression lines of Δ-changes of HDL (*x*-axis) and EPA, within experimental treatment arm (**a**) and control arm (**b**). Contour plots: joint distributions of Δ-changes of HDL (*x*-axis) and EPA, within experimental treatment arm (**c**) and control arm (**d**).

**Figure 3:**
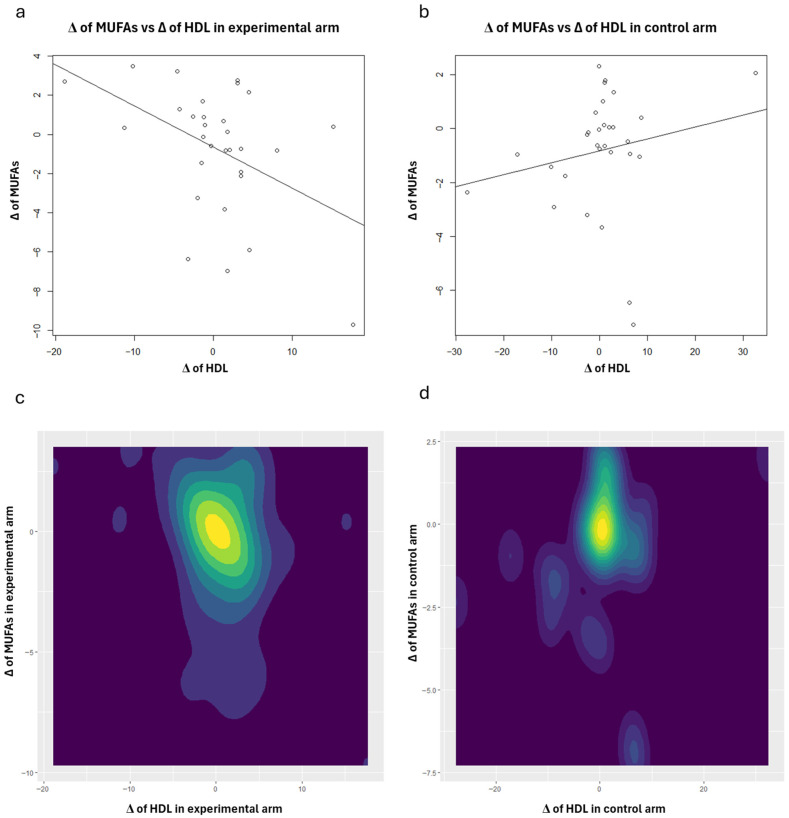
Scatter plots: joint distributions and regression lines of Δ-changes of HDL (*x*-axis) and MUFAs, within experimental treatment arm (**a**) and control arm (**b**). Contour plots: joint distributions of Δ-changes of HDL (*x*-axis) and MUFAs, within experimental treatment arm (**c**) and control arm (**d**).

**Figure 4:**
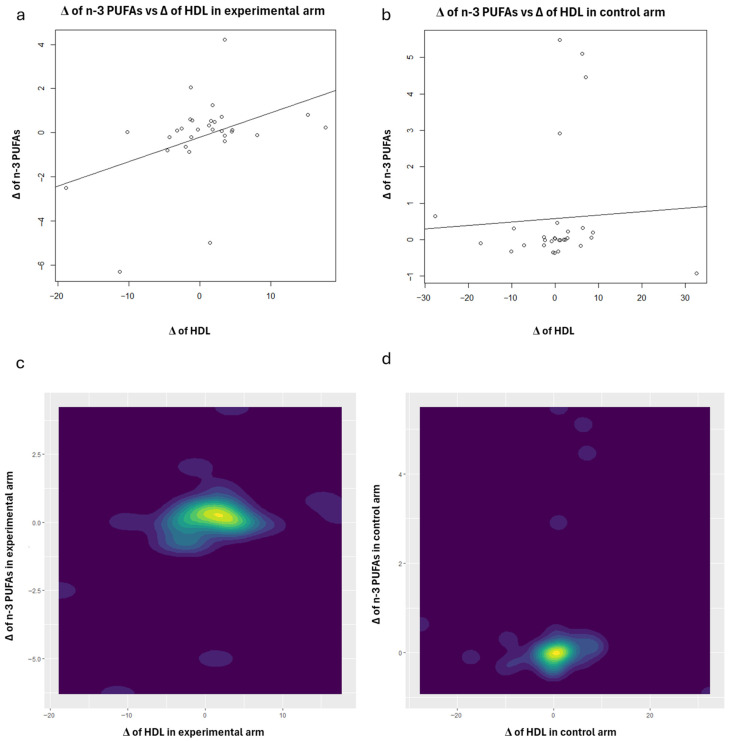
Scatter plots: joint distributions and regression lines of Δ-changes of HDL (*x*-axis) and *n*-3 PUFAs, within experimental treatment arm (**a**) and control arm (**b**). Contour plots: joint distributions of Δ-changes of HDL (*x*-axis) and *n*-3 PUFAs, within experimental treatment arm (**c**) and control arm (**d**).

**Figure 5:**
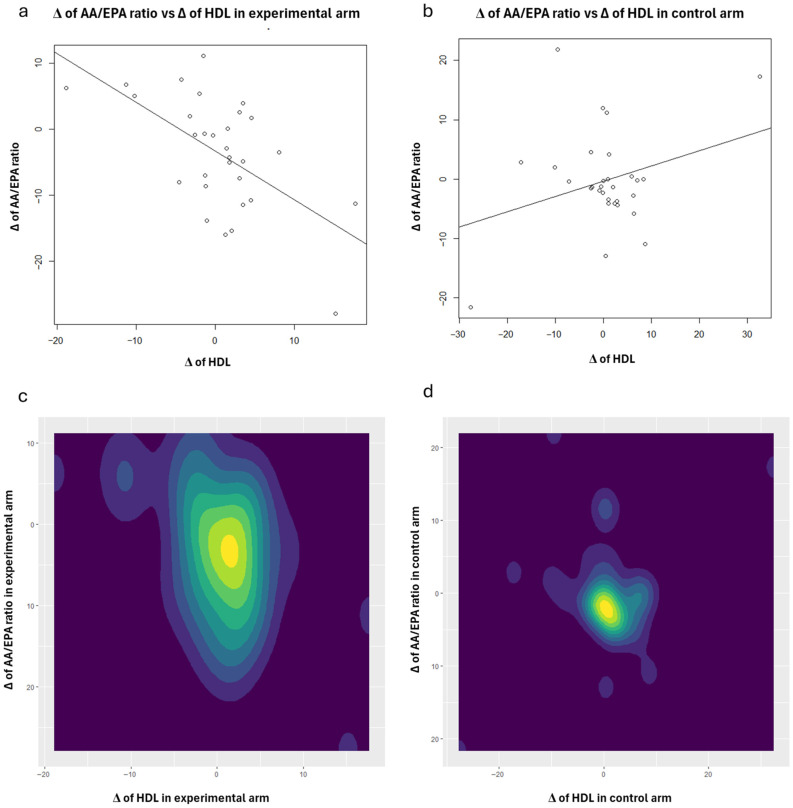
Scatter plots: joint distributions and regression lines of Δ-changes of HDL (*x*-axis) and AA/EPA ratio, within experimental treatment arm (**a**) and control arm (**b**). Contour plots: joint distributions of Δ-changes of HDL (*x*-axis) and AA/EPA ratio, within experimental treatment arm (**c**) and control arm (**d**).

To enhance the interpretation of the correlation analysis results concerning HDL, scatter plots (with regression lines) and contour plots were also generated (Figure 1, Figure 2, Figure 3, Figure 4 and Figure 5). Specifically, these plots illustrate the joint distribution between serum fatty acids and HDL Δ-changes by arm: a and b present the scatter plots that display the x-y points of the patients, while c and d show the contour plots, which represent the 2D kernel density estimations of the frequency of the x-y points.

In the experimental treatment arm, it is interesting to note that Oleic acid and HDL exhibit an inverse correlation; as Oleic acid decreases, HDL increases (Figure 1). A similar trend was observed with MUFAs (Figure 3) and the AA/EPA ratio (Figure 5). In contrast, we observed a positive correlation between EPA and *n*-3 PUFAs vs. HDL following the consumption of oranges, as shown in Figure 2 and Figure 4, respectively.

It is worth pointing out that the correlation analysis remains exploratory and should not be used to imply treatment effects or mechanisms.

Finally, post hoc analysis [29,30,36,37] was performed on the observed standardized mean differences of the Δ-changes of the Oleic acid, as reported in the Supplementary Material.

## 4. Discussion

Although this is a trial and further investigation should be done to better evaluate the effect of consuming polyphenol-rich “Navelina” oranges, here, we observed a potential association of “Navelina” orange polyphenols on serum fatty acid profiles in patients with MASLD. This clinical trial is one of the first to focus on the consumption of pulp tissue rather than orange juice. As previously published, the total polyphenol content in “Navelina” oranges is higher in the flesh tissue than in the juice [38]. The flesh tissue is covered by a membrane called albedo. Albedo is very rich in flavonoids such as hesperidin, which offers important antioxidants and anti-inflammatory properties.

**Figure 6:**
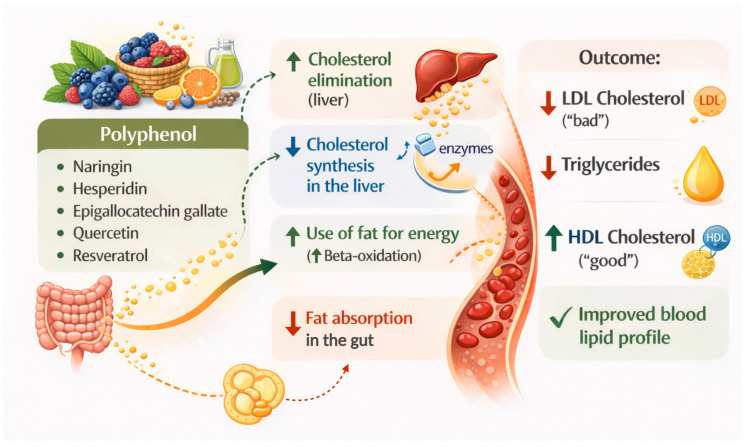
Role of polyphenols in modulating lipid metabolism. Image generated using ChatGPT (OpenAI, GPT-5.3) based on author-defined prompts and manually refined.

The literature indicates that polyphenol intake may modulate circulating lipid parameters [16,17,39]. Figure 6 illustrates the role of polyphenols in modulating lipid metabolism through multiple interconnected mechanisms involving intestinal absorption, hepatic processing, and peripheral lipid utilization. Flavanones are among the main bioactive compounds responsible for these effects, although their biological activity largely depends on their metabolic transformation [40]. At the intestinal level, polyphenols may contribute to a reduction in lipid absorption by interfering with micellar solubilization and lipid transport processes. This mechanism may lead to a lower delivery of dietary lipids to the liver, a reduction in cholesterol synthesis, and the inhibition of key enzymes. Furthermore, increased fatty acid oxidation and improved lipolysis may further support the improvement of lipid homeostasis. Stimulation of mitochondrial β-oxidation promotes fatty acid utilization, thus contributing to the reduction in circulating triglycerides [41]. The antioxidant and anti-inflammatory properties of polyphenols may further contribute to these findings. They can reduce oxidative stress and inflammatory signaling, improving lipoprotein function and reducing LDL oxidation, which is a key step in atherogenesis [42]. Nonetheless, it must be acknowledged that the extent of these benefits is generally modest and influenced by several factors, including the dose, dietary matrix, duration of intake, and basal metabolic state. Overall, polyphenols appear to modulate hepatic cholesterol synthesis and fatty acid utilization, while also attenuating oxidative stress and inflammatory processes. In accordance with the literature, our analysis reveals that polyphenols influence total serum cholesterol levels. This finding is evident from the GEE analysis, which shows a *p*-value for β_TxTR_ between 0.05 and 0.10, indicating a suggestive interaction.

In this study, a slight improvement in the serum lipidomic profile was observed after 4 weeks of dietary orange supplementation. Specifically, we detected a mild reduction in AA levels and a slight increase in EPA in the experimental group. Elevated AA levels have been strongly associated with a chronic low-grade inflammatory state, characteristic of obesity and metabolic liver diseases [43,44]. Conversely, several studies have demonstrated that EPA exerts protective effects against obesity, insulin resistance, and inflammatory processes [44].

Both AA and EPA belong to the PUFAs family; however, AA is an *n*-6 fatty acid with predominantly pro-inflammatory properties, while EPA is an *n*-3 fatty acid known for its anti-inflammatory and immunomodulatory effects. These fatty acids represent downstream products of the respective *n*-6 and *n*-3 PUFAs metabolic pathways, with Linoleic acid and alpha-Linolenic acid serving as their dietary precursors.

Consistent with these findings, the fatty acid ratio analysis revealed a moderate reduction in the AA/EPA ratio in the experimental treatment arm, although this reduction was not statistically significant. This ratio is considered a sensitive biomarker of nutritional status and inflammatory balance and closely reflects dietary fatty acid intake [45]. An elevated AA/EPA ratio, skewed toward AA dominance, has been associated with the development of several metabolic disorders, including obesity and NAFLD [46,47].

In this study, a significant correlation also emerged between the Δ-change of lipidomic parameters and the Δ-change of HDL cholesterol levels. In particular, we demonstrated the positive correlation between the Δ-change of *n*-3 PUFAs and HDL, both of which are protective markers for human health. HDL particles play a central role in reverse cholesterol transport and exhibit well-documented anti-inflammatory, antioxidant, and endothelial-protective functions [8]. Although these are only exploratory associations, as is well established in the literature, increased levels of these protective markers are indicative of an improvement in MASLD status [8]. In fact, numerous dietary components, particularly polyphenols and unsaturated fatty acids, have been shown to rapidly influence both HDL concentration and functionality by enhancing cholesterol efflux capacity, modulating apolipoprotein A-I expression, and improving HDL antioxidant properties [48]. Correlation analysis of the control group revealed statistically significant inverse correlations between Oleic acid (r = −0.463, *p* = 0.010) and MUFAs (r = −0.402, *p* = 0.028) vs. total cholesterol. This suggests that higher levels of Oleic acid are associated with lower levels of total cholesterol. While this finding supports the beneficial effects of MUFAs on lipid profiles, it should be noted that, as it is based on a correlational analysis in the absence of intervention, it does not permit the establishment of a causal relationship and may be influenced by confounding factors.

## 5. Conclusions

Finally, this study also presents inevitable limitations. From a methodological perspective, the small sample size per arm and short 4-week duration limit generalizability and causal claims. Finally, it is worth pointing out that the number of participants included in the trial is too small. In addition, the nature of this clinical trial is explorative, and no formal multiplicity strategy was implemented to manage multiple testing on multiple Spearman correlations; therefore, the results should be interpreted with caution. Regarding that, once more, these results are not conclusive and represent only exploratory associations.

To our knowledge, this study represents one of the first clinical investigations into the effect of “Navelina” orange polyphenols on serum fatty acid profiles in patients with MASLD. In addition, the study may serve as the basis for further research into the nutraceutical effects of oranges on lipid metabolism, specifically examining the relationship between the serum lipidomic profile and markers of inflammation or oxidative stress. Finally, future research will also focus on developing a dose–response curve to strengthen the health benefits of orange consumption and further consolidate the findings.

## Figures and Tables

**Table 1:** Baseline characteristics of the sample.

Variable	Baseline		
	Experimental Arm (n = 30)	Control Arm (n = 30)	
	n (%) or median [IQR]	n (%) or median [IQR]	*p*-value
Demographic and lifestyle parameters			
Sex (M/F)	23/7 (76.766/23.333)	20/10 (66.667/33.333)	0.567
Age (years)	54.500 (44.250; 60.750)	52.000 (44.500; 58.500)	0.584
Anthropometric and clinical parameters			
Weight (kg)	91.900 (85.250; 97.875)	89.300 (85.975; 99.500)	0.717
BMI (kg/m^2^)	31.550 (29.350; 35.303)	31.100 (28.750; 35.100)	0.679
Dietary intake			
Total Proteins (g)	93.610 (80.985; 106.955)	87.735 (75.527; 94.480)	*0.069*
Total Lipids (g)	94.455 (84.800; 108.287)	85.390 (76.257; 93.477)	**0.044**
Total carbohydrates (g)	194.395 (177.090; 246.070)	237.800 (209.828; 248.178)	*0.062*
Calorie (Kcal)	2074.735 (1887.170; 2189.952)	1969.615 (1879.470; 2090.302)	0.283
Biochemical parameters			
Total cholesterol (mg/dL)	207.000 (185.000; 221.750)	172.000 (157.750; 211.000)	**0.048**
HDL cholesterol (mg%)	45.150 (36.850; 52.700)	51.450 (45.575; 55.925)	*0.073*
LDL cholesterol (mg/dL)	136.950 (112.925; 155.300)	116.050 (93.780; 145.030)	*0.093*
sdLDL score (%)	1.455 (0.000; 7.077)	0.000 (0.000; 4.243)	0.449
VLDL (mg/dL)	33.000 (28.250; 36.500)	28.000 (23.500; 38.500)	0.155
IDL-C (mg/dL)	24.000 (17.010; 28.500)	18.001 (13.500; 24.002)	0.108
IDL-B (mg/dL)	10.500 (7.250; 13.750)	10.005 (7.250; 12.000)	0.947
IDL-A (mg/dL)	19.007 (13.012; 25.250)	17.500 (12.250; 24.250)	0.563
LDL-1 (mg/dL)	43.509 (32.251; 50.012)	36.005 (28.094; 41.751)	*0.069*
LDL-2 (mg/dL)	18.500 (13.021; 28.250)	17.006 (11.004; 25.101)	0.524
LDL-3 (mg/dL)	1.032 (0.001; 4.508)	0.000 (0.000; 2.750)	0.454
LDL-4 (mg/dL)	0.000 (0.000; 0.000)	0.000 (0.000; 0.000)	0.597

Note. Data are n (%) or median [Interquartile range, IQR]. The *p*-values by the Mann–Whitney test. In bold, the significant results (*p* < 0.05); in italics, (0.05 < *p* < 0.10) trends. Abbreviations: BMI, Body Mass Index; HDL, High-Density Lipoprotein; LDL, Low-Density Lipoprotein; sdLDL, small dense Low-Density Lipoprotein; VLDL, Very Low-Density Lipoprotein; IDL, Intermediate Density Lipoprotein.

**Table 2:** Outcomes per treatment with Generalized Estimating Equation (GEE) output of the expected effects (β).

Outcome	Experimental Arm		Control Arm		GEE		
	Baseline	Follow Up	Baseline	Follow Up	β_T_ *p*-Value 95%CI	β_TR_ *p*-Value 95%CI	β_TxTR_ *p*-Value 95%CI
Palmitic acid (%)	17.171 (16.200; 18.600)	16.495 (15.700; 17.500)	17.391 (16.700; 18.900)	17.100 (16.300; 17.700)	−0.579 0.175 −1.416; 0.258	*−0.806* *0.052* *−1.621*; *0.007*	−0.152 0.803 −1.349; 1.044
cis-9-Palmitoleic acid (%)	0.765 (0.625; 1.002)	0.805 (0.568; 0.970)	0.900 (0.688; 1.398)	0.779 (0.672; 1.092)	−0.124 0.201 −0.314; 0.066	−0.146 0.174 −0.356; 0.064	0.107 0.432 −0.161; 0.377
Stearic acid (%)	7.042 (6.360; 7.710)	6.823 (6.350; 7.380)	6.890 (6.290; 7.160)	6.744 (6.270; 7.280)	0.130 0.619 −0.385; 0.647	**0.554** **0.024** **0.071; 1.036**	−0.341 0.347 −1.052; 0.370
trans-9-Elaidic acid (%)	0.510 (0.335; 0.690)	0.476 (0.272; 0.703)	0.540 (0.412; 0.680)	0.454 (0.308; 0.692)	−0.084 0.150 −0.200; 0.031	−0.057 0.440 −0.204; 0.089	0.056 0.570 −0.137; 0.251
Oleic acid (%)	16.037 (14.400; 18.400)	15.962 (13.400; 17.800)	15.624 (14.800; 17)	15.416 (13.600; 17.500)	−0.655 0.290 −1.859; 0.549	−0.185 0.810 −1.727; 1.357	−0.011 0.990 −2.008; 1.986
Linolelaidic acid (%)	0.040 (0.022; 0.057)	0.039 (0.022; 0.060)	0.048 (0.040; 0.067)	0.051 (0.040; 0.067)	0.003 0.465 −0.005; 0.012	*−0.004* *0.335* *−0.014*; *0.004*	−0.004 0.466 −0.017; 0.007
LA (%)	45.697 (39.200; 48)	46.992 (42.900; 50.400)	48.472 (44.300; 49.700)	48.206 (45; 50.500)	0.354 0.784 −2.179; 2.887	−1.535 0.306 −4.474; 1.404	2.418 0.203 −1.301; 6.139
γ-Linolenic acid (%)	0.958 (0.180; 1.760)	0.969 (0.250; 1.970)	0.403 (0.180; 1.040)	0.647 (0.270; 1.460)	0.298 * 0.580 −0.762; 1.358	0.301 * 0.590 −0.806; 1.409	−0.498 * 0.470 −1.859; 0.862
ALA (%)	0.093 (0.040; 0.160)	0.067 (0.020; 0.178)	0.010 (0.010; 0.020)	0.013 (0.010; 0.028)	0.002 0.427 −0.003; 0.009	**0.096** **<0.001** **0.053; 0.139**	−0.005 0.868 −0.067; 0.057
Arachidic acid (%)	0.077 (0.053; 0.097)	0.074 (0.060; 0.080)	0.065 (0.050; 0.080)	0.060 (0.042; 0.080)	0.001 0.916 −0.011; 0.012	*0.016* *0.009* *0.004*; *0.027*	−0.001 0.908 −0.018; 0.016
cis-8,11,14-Eicosatrienoic acid (%)	1.622 (1.39; 2)	1.553 (1.36; 1.78)	1.708 (1.21; 2.29)	1.723 (1.20; 2.21)	0.072 0.638 −0.230; 0.375	0.076 0.560 −0.181; 0.335	−0.203 0.259 −0.556; 0.1501
AA (%)	5.213 (4.500; 5.800)	4.588 (4.170; 5.260)	4.297 (3.580; 5.140)	4.447 (3.820; 4.810)	−0.005 0.982 −0.500; 0.489	**1.011** **<0.001** **0.443; 1.578**	−0.411 0.247 −1.106; 0.285
cis-11,14,17-Eicosatrienoicacid (%)	0.070 (0.050; 0.100)	0.064 (0.050; 0.080)	0.049 (0.040; 0.060)	0.053 (0.040; 0.090)	0.535 * **0.033** **0.043; 1.026**	0.437 * 0.167 −0.182; 1.058	*−0.728* * 0.060 *−1.488*; *0.031*
Behenic acid (%)	0.025 (0.020; 0.030)	0.025 (0.013; 0.030)	0.022 (0.020; 0.270)	0.026 (0.013; 0.500)	0.000 0.937 −0.000; 0.000	−0.011 0.148 −0.025; 0.003	0.000 0.989 −0.006; 0.006
Erucic acid (%)	0.048 (0.032; 0.068)	0.050 (0.040; 0.070)	0.053 (0.040; 0.070)	0.052 (0.023; 0.070)	−0.021 0.210 −0.055; 0.011	−0.016 0.390 −0.053; 0.021	0.024 0.190 −0.011; 0.060
EPA (%)	0.301 (0.210; 0.530)	0.340 (0.260; 0.470)	0.242 (0.170; 0.305)	0.232 (0.172; 0.408)	−0.001 0.986 −0.129; 0.126	*0.204* *0.057* *−0.006*; *0.415*	0.070 0.606 −0.198; 0.339
Lignoceric acid (%)	0.125 (0.102; 0.185)	0.117 (0.080; 0.158)	0.104 (0.082; 0.157)	0.080 (0.062; 0.108)	**−0.033** **0.040** **−0.022; 0.046**	**0.031** **0.002** **0.001; 0.061**	0.012 0.488 −0.022; 0.046
DHA (%)	0.918 (0.715; 1.308)	0.869 (0.682; 1.118)	0.775 (0.647; 0.995)	0.740 (0.585; 0.955)	0.014 0.873 −0.159; 0.187	*0.197* *0.051* *−0.001*; *0.395*	−0.029 0.822 −0.284; 0.226
SFAs (%)	26.366 (25.500; 28.200)	25.112 (24; 26.300)	26.328 (24.500; 27.600)	25.615 (24; 27.500)	−0.447 0.484 −1.700; 0.805	−0.185 0.738 −1.271; 0.900	−0.744 0.376 −2.396; 0.907
MUFAs (%)	17.843 (15.630; 19.800)	17.524 (14.620; 19.550)	17.414 (16.610; 19.510)	17.345 (15.140; 18.910)	−0.868 0.217 −2.249; 0.512	−0.325 0.713 −2.063; 1.412	0.157 0.890 −2.082; 2.396
PUFAs (%)	55.843 (51.800; 58.900)	57.459 (53.500; 60.400)	56.203 (53.400; 58)	56.679 (54.800; 59.400)	1.248 0.231 −0.795; 3.291	0.490 0.682 −1.856; 2.837	0.755 0.645 −2.463; 3.974
*n*-3 PUFAs (%)	1.478 (1.110; 2.090)	1.393 (1.130; 1.900)	1.115 (0.910; 1.470)	1.085 (0.950; 1.620)	0.370 0.135 −0.115; 0.856	**0.919** **0.010** **0.210; 1.627**	−0.523 0.214 −1.351; 0.303
AA/EPA ratio (u)	18.098 (9.900; 25.600)	13.710 (9.800; 19.100)	20.784 (12.900; 24.900)	20.219 (10.300; 25.300)	−0.287 0.909 −5.246; 4.671	−0.855 0.693 −5.111; 3.400	−3.299 0.304 −9.592; 2.993
Total cholesterol (mg/dL)	207.000 (185; 222)	193.500 (166; 217)	172.000 (158; 211)	175.000 (162; 212)	**7.987** **0.043** **0.234; 15.740**	*0.444* *0.857* *−4.397 5.286*	*−11.383* *0.060* *−23.269*; *0.501*
LDL (mg/dL)	136.950 (112.900; 155.300)	126.300 (108.300; 157.900)	116.050 (93.800; 145)	113.70 (93.100; 152.100)	8.138 0.111 −1.862; 18.139	−2.504 0.554 −10.800 5.791	−5.798 0.416 −19.775; 8.178
HDL (mg%)	45.150 (36.850; 52.700)	47.400 (40.520; 51.350)	51.450 (45.580; 55.920)	48.500 (45.300; 55.900)	0.069 0.981 −5.495; 5.633	*−5.327* *0.079* *−11.271*; *0.617*	0.886 0.829 −7.152; 8.924
sdLDL score (%)	1.453 (0.000; 7.080)	0.000 (0.000; 3.830)	0.000 (0.000; 4.240)	0.000 (0.000; 2.630)	−0.519 * 0.626 −2.609; 1.570	0.277 * 0.785 −1.725; 2.281	−0.547 * 0.701 −3.338; 2.244

Note. Dates are median [Interquartile range, IQR]. GEE: Generalized Estimating Equation (Gamma distribution, link = identity). The models’ time parameters (β_T_) are interpreted as average time changes of the outcomes between the two separate measurements (follow-up—baseline measurements); the parameters associated with the treatment (β_TR_) are interpreted as average baseline differences between the two treatments (the control one represents the reference category). The interaction parameters (β_TxTR_) are interpreted as average time differences of the outcomes (from baseline) between treatments. 95% CI: 95% confidence interval. In bold, the significant results (*p* < 0.05), in italics, (0.05 < *p* < 0.10) trends. * Results achieved by Gaussian GEE. Abbreviations: LA, Linoleic acid; ALA, Alpha-Linolenic acid; AA, Arachidonic acid; EPA, Eicosapentaenoic acid; DHA, Docosahexaenoic acid; SFAs, Saturated Fatty Acids; MUFAs, Monounsaturated Fatty Acids; PUFAs, Polyunsaturated Fatty Acids; *n*-3 PUFAs, omega-3 Polyunsaturated Fatty Acids; AA/EPA ratio, Arachidonic acid/Eicosapentaenoic acid ratio; LDL, Low-Density Lipoprotein; HDL, High- Density Lipoprotein; sdLDL, small dense Low-Density Lipoprotein.

**Table 3:** Δ-changes in Spearman correlations between serum fatty acids and biochemical markers stratified by arm.

r (*p*-Value)	Total Cholesterol	HDL	LDL
Experimental arm			
Oleic acid	−0.258 0.168	**−0.368** **0.046**	−0.278 0.137
EPA	**0.386** **0.035**	**0.447** **0.013**	0.240 0.202
MUFAs	−0.227 0.227	**−0.384** **0.036**	−0.233 0.214
*n*-3 PUFAs	*0.325* *0.080*	**0.403** **0.027**	0.160 0.398
AA/EPA ratio	*−0.325* *0.080*	**−0.522** **0.003**	−0.167 0.377
Control arm			
Oleic acid	**−0.463** **0.010**	0.251 0.181	−0.259 0.166
EPA	−0.054 0.776	0.280 0.134	0.175 0.355
MUFAs	**−0.402** **0.028**	0.243 0.195	−0.224 0.234
*n*-3 PUFAs	−0.037 0.845	0.250 0.183	−0.123 0.517
AA/EPA ratio	0.010 0.958	−0.180 0.342	−0.212 0.262

Note. r: Spearman correlation; *p*: *p*-value. In bold, the significant results (*p* < 0.05), in italics, (0.05 < *p* < 0.10) trends. Abbreviations: HDL, High-Density Lipoprotein; LDL, Low-Density Lipoprotein; EPA, Eicosapentaenoic acid; MUFAs: Monounsaturated fatty acids; *n*-3 PUFAs, omega-3 Polyunsaturated Fatty Acids; AA/EPA ratio, Arachidonic acid/Eicosapentaenoic Acid ratio.

## Data Availability

Data are available from the corresponding author upon reasonable requests.

## Supplementary Materials

The following supporting information can be downloaded at: https://www.mdpi.com/article/10.3390/nu18081254/s1. Figure S1: Plot of the post hoc power analysis for Δ-change * between arms of the Oleic acid.

## Abbreviations

The following abbreviations are used in this manuscript:

MASLD	Metabolic Dysfunction-Associated Steatotic Liver Disease
NAFLD	Non-Alcoholic Fatty Liver Disease
GC	Gas Chromatography
FID	Flame Ionization Detector
LC	Liquid Chromatography
MS	Mass Spectrometry
SFAs	Saturated Fatty Acids
MUFAs	Monounsaturated Fatty Acids
PUFAs	Polyunsaturated Fatty Acids
*n*-3	Omega-3
*n*-6	Omega-6
LDL	Low-Density Lipoprotein
HDL	High-Density Lipoprotein
VLDL	Very Low-Density Lipoprotein
IDL	Intermediate Density Lipoprotein
ALA	Alpha-Linolenic acid
EPA	Eicosapentaenoic acid
DHA	Docosahexaenoic acid
LA	Linoleic acid
AA	Arachidonic acid
AA/EPA ratio	Arachidonic acid/Eicosapentaenoic acid ratio
SCD1	Stearoyl-CoA Desaturase-1
CAP	Controlled Attenuation Parameter
BMI	Body Mass Index
sdLDL	Small Dense Low-Density Lipoprotein
IQR	Interquartile range
GEE	Generalized Estimating Equation
SMD	Standardized Mean Difference

## References

[1] Wong V.W., Wong G.L., Woo J., Abrigo J.M., Chan C.K., Shu S.S., Leung J.K., Chim A.M., Kong A.P., Lui G.C. (2021). Impact of the New Definition of Metabolic Associated Fatty Liver Disease on the Epidemiology of the Disease. Clin. Gastroenterol. Hepatol..

[2] Eslam M., Newsome P.N., Sarin S.K., Anstee Q.M., Targher G., Romero-Gomez M., Zelber-Sagi S., Wai-Sun Wong V., Dufour J.F., Schattenberg J.M. (2020). A new definition for metabolic dysfunction-associated fatty liver disease: An international expert consensus statement. J. Hepatol..

[3] Boccatonda A., Andreetto L., D’Ardes D., Cocco G., Rossi I., Vicari S., Schiavone C., Cipollone F., Guagnano M.T. (2023). From NAFLD to MASLD: Definition, Pathophysiological Basis and Cardiovascular Implications. Biomedicines.

[4] Mantovani A., Scorletti E., Mosca A., Alisi A., Byrne C.D., Targher G. (2020). Complications, morbidity and mortality of nonalcoholic fatty liver disease. Metabolism.

[5] Notarnicola M., Tutino V., De Nunzio V., Cisternino A.M., Cofano M., Donghia R., Giannuzzi V., Zappimbulso M., Milella R.A., Giannelli G. (2024). Daily Orange Consumption Reduces Hepatic Steatosis Prevalence in Patients with Metabolic Dysfunction-Associated Steatotic Liver Disease: Exploratory Outcomes of a Randomized Clinical Trial. Nutrients.

[6] Tatoli R., Caterina B., Donghia R., Pesole P.L., Fontana L., Giannelli G. (2025). Dietary Omega-3 Fatty Acids from Fish and Risk of Metabolic Dysfunction-Associated Steatotic Liver Disease in a Mediterranean Population: Findings from the NUTRIHEP Cohort. Nutrients.

[7] Heeren J., Scheja L. (2021). Metabolic-associated fatty liver disease and lipoprotein metabolism. Mol. Metab..

[8] Cui D., Yu X., Guan Q., Shen Y., Liao J., Liu Y., Su Z. (2025). Cholesterol metabolism: Molecular mechanisms, biological functions, diseases, and therapeutic targets. Mol. Biomed..

[9] Sirtori C.R., Fumagalli R. (2006). LDL-cholesterol lowering or HDL-cholesterol raising for cardiovascular prevention. A lesson from cholesterol turnover studies and others. Atherosclerosis.

[10] Sun Y., Saito K., Saito Y. (2022). Lipidomic Analysis of Extracellular Vesicles Isolated from Human Plasma and Serum. Methods Mol. Biol..

[11] Gehin C., Fowler S.J., Trivedi D.K. (2023). Chewing the fat: How lipidomics is changing our understanding of human health and disease in 2022. Anal. Sci. Adv..

[12] Ding M., Rexrode K.M. (2020). A Review of Lipidomics of Cardiovascular Disease Highlights the Importance of Isolating Lipoproteins. Metabolites.

[13] Hliwa A., Ramos-Molina B., Laski D., Mika A., Sledzinski T. (2021). The Role of Fatty Acids in Non-Alcoholic Fatty Liver Disease Progression: An Update. Int. J. Mol. Sci..

[14] Zhou H., Urso C.J., Jadeja V. (2020). Saturated Fatty Acids in Obesity-Associated Inflammation. J. Inflamm. Res..

[15] Garcia-Martinez I., Alen R., Pereira L., Povo-Retana A., Astudillo A.M., Hitos A.B., Gomez-Hurtado I., Lopez-Collazo E., Bosca L., Frances R. (2023). Saturated fatty acid-enriched small extracellular vesicles mediate a crosstalk inducing liver inflammation and hepatocyte insulin resistance. JHEP Rep..

[16] Assy N., Nassar F., Nasser G., Grosovski M. (2009). Olive oil consumption and non-alcoholic fatty liver disease. World J. Gastroenterol..

[17] Saini R.K., Keum Y.S. (2018). Omega-3 and omega-6 polyunsaturated fatty acids: Dietary sources, metabolism, and significance—A review. Life Sci..

[18] Malarvannan M., Sabavath B.T.N., Gaddam V., Paul D. (2025). Transformative potentials, challenges and innovative solutions of lipidomics in multiple clinical applications. Talanta.

[19] Dhibi M., Brahmi F., Mnari A., Houas Z., Chargui I., Bchir L., Gazzah N., Alsaif M.A., Hammami M. (2011). The intake of high fat diet with different trans fatty acid levels differentially induces oxidative stress and non alcoholic fatty liver disease (NAFLD) in rats. Nutr. Metab..

[20] Notarnicola M., De Nunzio V., Lippolis T., Tutino V., Cisternino A.M., Iacovazzi P.A., Milella R.A., Gasparro M., Negro R., Polignano M. (2022). Beneficial Effects of Table Grape Use on Serum Levels of Omega-3 Index and Liver Function: A Randomized Controlled Clinical Trial. Biomedicines.

[21] Rasouli H., Farzaei M.H., Khodarahmi R. (2017). Polyphenols and their benefits: A review. Int. J. Food Prop..

[22] Lippolis T., Cofano M., Caponio G.R., De Nunzio V., Notarnicola M. (2023). Bioaccessibility and Bioavailability of Diet Polyphenols and Their Modulation of Gut Microbiota. Int. J. Mol. Sci..

[23] Tutino V., Gigante I., Scavo M.P., Refolo M.G., Nunzio V., Milella R.A., Caruso M.G., Notarnicola M. (2020). Stearoyl-CoA Desaturase-1 Enzyme Inhibition by Grape Skin Extracts Affects Membrane Fluidity in Human Colon Cancer Cell Lines. Nutrients.

[24] Cofano M., Saponara I., De Nunzio V., Pinto G., Aloisio Caruso E., Centonze M., Notarnicola M. (2025). Hesperidin Is a Promising Nutraceutical Compound in Counteracting the Progression of NAFLD In Vitro. Int. J. Mol. Sci..

[25] Yan J., Fine J. (2004). Estimating equations for association structures. Stat. Med..

[26] Shults J., Hilbe J. (2014). Quasi-Least Squares Regression.

[27] Locascio J.J., Atri A. (2011). An overview of longitudinal data analysis methods for neurological research. Dement. Geriatr. Cogn. Dis. Extra.

[28] Shoukri M.M. (2018). Analysis of Correlated Data with SAS and R.

[29] Cohen J. (1988). Statistical Power Analysis for the Behavioral Sciences.

[30] Cohen J. (1990). Things I have learned (so far). Am. Psychol..

[31] Højsgaard S., Halekoh U., Yan J. (2006). The R Package geepack for Generalized Estimating Equations. J. Stat. Softw..

[32] Yan J. (2002). Geepack: Yet Another Package for Generalized Estimating Equations. R-News.

[33] Harrell J.R. (2024). Hmisc: Harrell Miscellaneous. R Package Version 5.2-0. https://CRAN.R-project.org/package=Hmisc.

[34] Champely S. (2020). _pwr: Basic Functions for Power Analysis_. R Package Version 1.3-0. https://CRAN.R-project.org/package=pwr.

[35] Wickham H. (2016). ggplot2: Elegant Graphics for Data Analysis.

[36] Crespi C.M. (2020). Power and Sample Size in R.

[37] Hoening J.M., Heisey D.M. (2001). The Abuse of Power: The Pervasive Fallacy of Power Calculations for Data Analysis. Am. Stat..

[38] Saponara I., Caruso E.A., Cofano M., De Nunzio V., Pinto G., Centonze M., Notarnicola M. (2025). Anti-Inflammatory and Anti-Fibrotic Effects of a Mixture of Polyphenols Extracted from “Navelina” Orange in Human Hepa-RG and LX-2 Cells Mediated by Cannabinoid Receptor 2. Int. J. Mol. Sci..

[39] Zupo R., Castellana F., Crupi P., Desantis A., Rondanelli M., Corbo F., Clodoveo M.L. (2023). Olive Oil Polyphenols Improve HDL Cholesterol and Promote Maintenance of Lipid Metabolism: A Systematic Review and Meta-Analysis of Randomized Controlled Trials. Metabolites.

[40] Rudrapal M., Rakshit G., Singh R.P., Garse S., Khan J., Chakraborty S. (2024). Dietary Polyphenols: Review on Chemistry/Sources, Bioavailability/Metabolism, Antioxidant Effects, and Their Role in Disease Management. Antioxidants.

[41] Wei H., Rui J., Yan X., Xu R., Chen S., Zhang B., Wang L., Zhang Z., Zhu C., Ma M. (2025). Plant polyphenols as natural bioactives for alleviating lipid metabolism disorder: Mechanisms and application challenges. Food Res. Int..

[42] Scavuzzi B.M., Dichi I. (2025). Effects of a Diet Rich in Polyphenols on Lipid Metabolism: An Updated Narrative Review. Heart Mind.

[43] Ma Y., Jiang J., Zhao C., Wei B., Gao J. (2025). Arachidonic acid metabolism in metabolic dysfunction-associated steatotic liver disease and liver fibrosis. Hepatol. Commun..

[44] Calder P.C. (2013). Omega-3 polyunsaturated fatty acids and inflammatory processes: Nutrition or pharmacology?. Br. J. Clin. Pharmacol..

[45] Simopoulos A.P. (2006). Evolutionary aspects of diet, the omega-6/omega-3 ratio and genetic variation: Nutritional implications for chronic diseases. Biomed. Pharmacother..

[46] Tutino V., De Nunzio V., Caruso M.G., Veronese N., Lorusso D., Di Masi M., Benedetto M.L., Notarnicola M. (2019). Elevated AA/EPA Ratio Represents an Inflammatory Biomarker in Tumor Tissue of Metastatic Colorectal Cancer Patients. Int. J. Mol. Sci..

[47] Simopoulos A.P., DiNicolantonio J.J. (2016). The importance of a balanced omega-6 to omega-3 ratio in the prevention and management of obesity. Open Heart.

[48] Baba S., Osakabe N., Kato Y., Natsume M., Yasuda A., Kido T., Fukuda K., Muto Y., Kondo K. (2007). Continuous intake of polyphenolic compounds containing cocoa powder reduces LDL oxidative susceptibility and has beneficial effects on plasma HDL-cholesterol concentrations in humans. Am. J. Clin. Nutr..

