# Supplementary material for: Effect of 4-Week Consumption of “Navelina” Oranges on Serum Lipid Profile in Patients with MASLD: Evidence from a Randomized Clinical Trial
Source: Nutrients. 2026 Apr 16;18(8):1254. doi: 10.3390/nu18081254 (PMC13118749; doi:10.3390/nu18081254)
Supplement: Supplementary file 1 [file nutrients-18-01254-s001.zip › nutrients-4199380-supplementary.pdf]

Supplementary

Post hoc power analysis

We performed a post hoc power analysis using the Cohen's d (i. e., the standardized mean difference, SMD, of the delta changes, follow up-baseline), as effect size [1,2], on Oleic acid levels, a relevant MUFA in human serum. The post hoc power analysis is an estimate of the power of a test given the observed effect size and sample size. The underlying idea is to show that a non-significant result occurred because the power is insufficient [3]. Regarding this, to elicit all the eligible sample size values, we investigated the performance of post hoc power analysis by a simulation study, by varying the power values (x-axis) and achieving the corresponding sample sizes (y-axis), in relation to the observed effect size; the two-sided Type-I-error level was set at 0.05. However, it is worth noting that the post hoc power analysis is criticized, as has been well argued by Hoening & Heisey [4].

After calculations, the post hoc analysis returned a power value equal to 0.073, on the observed standardized mean differences of the Δ-changes of the Oleic acid (SMD=0.117). Next, the simulation study determined the sample sizes based on power values, allowing the comparison with the power achieved in this study (Figure S1).

Figure S1. Plot of the post hoc power analysis for Δ-change* between arms of the Oleic acid.

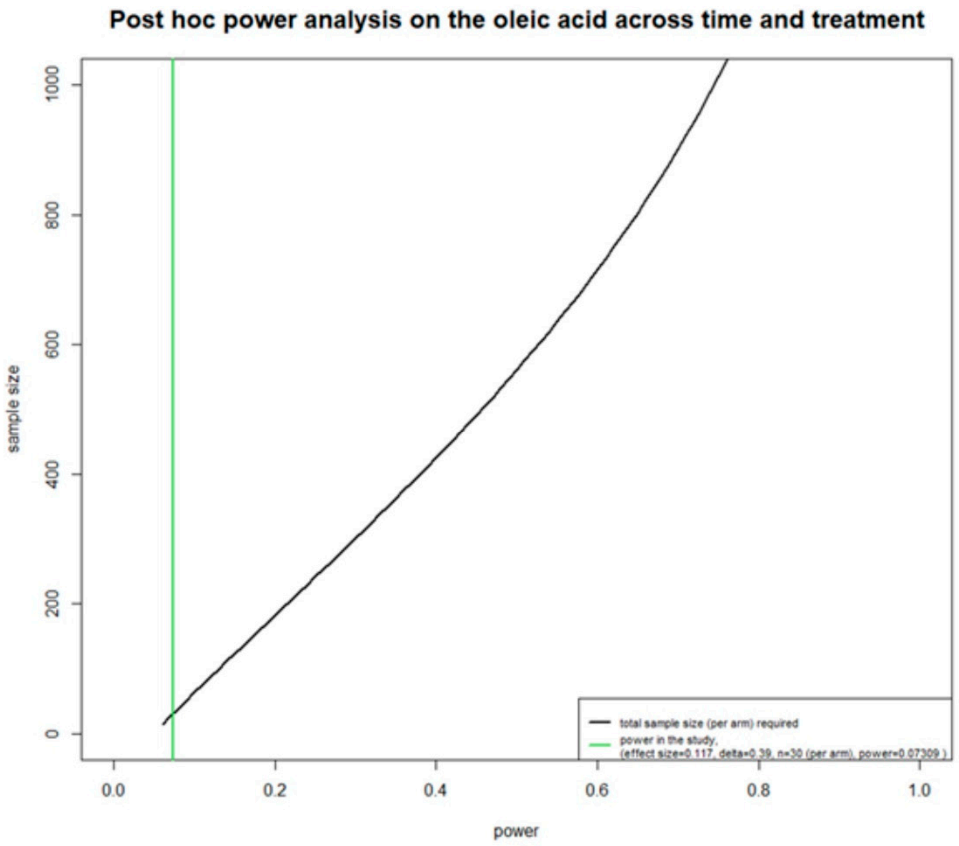

Note. * $\Delta_{\text{Experimental treatment Arm}} - \Delta_{\text{Control Arm}}$

References

[1] Cohen, J. Statistical Power Analysis for the Behavioral Sciences, 2nd ed.; Academic Press: New York, NY, USA, 1988. 525

[2] Cohen, J. Things I have learned (so far). *Am. Psychol.* **1990**, *45*, 1304–1312.

[3] Crespi, C.M. Power and Sample Size in R; Chapman & Hall: Boca Raton, FL, USA, **2020**; ISBN 9781138591622

[4] Hoening, J.M.; Heisey, D.M. The Abuse of Power: The Pervasive Fallacy of Power Calculations for Data Analysis. *Am. Stat.* **2001**, *55*, 19–24.
